# Meditation as an Adjunct to the Management of Acute Pain

**DOI:** 10.1007/s11916-023-01119-0

**Published:** 2023-06-07

**Authors:** Flavia Wipplinger, Niels Holthof, Lukas Andereggen, Richard D. Urman, Markus M. Luedi, Corina Bello

**Affiliations:** 1 Department of Anaesthesiology and Pain Medicine, Inselspital, Bern University Hospital, University of Bern, 3010 Freiburgstrasse Bern, Switzerland; 2 Department of Neurosurgery, Cantonal Hospital of Aarau, 5001 Aarau, Switzerland; 3 Department of Anaesthesiology, College of Medicine, The Ohio State University, Columbus, OH 43210 USA; 4 Department of Anaesthesiology and Pain Medicine, Cantonal Hospital of St, 9007 GallenSt. Gallen, Switzerland

**Keywords:** Acute pain, Multimodal treatment, Mindfulness, Meditation

## Abstract

**Purpose of Review:**

We aim to present current understanding and evidence for meditation, mostly referring to mindfulness meditation, for the management of acute pain and potential opportunities of incorporating it into the acute pain service practice.

**Recent Findings:**

There is conflicting evidence concerning meditation as a remedy in acute pain. While some studies have found a bigger impact of meditation on the emotional response to a painful stimulus than on the reduction in actual pain intensities, functional Magnet Resonance Imaging has enabled the identification of various brain areas involved in meditation-induced pain relief.

**Summary:**

Potential benefits of meditation in acute pain treatment include changes in neurocognitive processes. Practice and Experience are necessary to induce pain modulation. In the treatment of acute pain, evidence is emerging only recently. Meditative techniques represent a promising approach for acute pain in various settings.

## Introduction

Meditation is a contemplative practice that found its origins in Buddhism thousands of years ago [1••]. Over the past 40 years, these Buddhist traditions have made their way into the secular world as a way of promoting calmness and mental well-being [1••, 2••, 3]. Western medicine has also become increasingly interested in the potential benefits of meditation on mental and physical health [4] and the American Heart Association (AHA) states in their scientific statement on psychological health, well-being, and the mind-heart-body connection that “there is increasing evidence that psychological health may be causally linked to biological processes and behaviors that contribute to and cause (cardiovascular) disease” [5].

Meditation in a modern sense has been defined as “a family of self-regulation practices that focus on training attention and awareness in order to bring mental processes under greater voluntary control” [3]. Three common forms of meditation are focused attention meditation, mindfulness or open-monitoring meditation, and loving kindness or compassion meditation (Table 1). Mindfulness meditation has received much scientific attention in recent years, as it pursues a non-reactive form of sensory awareness [1••]. Mindfulness has been defined as “the awareness that emerges through paying attention on purpose, in the present moment, and nonjudgmentally to the unfolding of experience moment by moment” [2••]. During mindfulness practice, the practitioner observes their surroundings and monitors their sensations and internal dialogue, without becoming overly preoccupied with these perceptions. In this way, awareness of unpleasant sensations decreases the emotional response, an important notion that can be applied to understand the influence of mindfulness meditation on pain perception [1••].

**Table 1:** Common types of meditation

	**Focus**	**Aim**
**Focused attention meditation**	• Selective attention on a single object (typically breathing) [1••, 6] • *Mantra meditation*: reciting a word or phrase (often derived from Sanskrit roots) [7, 8]	• Recognizing mind wandering and restoring attention to the object of focus [1••, 6, 8], thereby o Calming the mind and reducing distractions [6] o Developing attentional control and monitoring skills [6, 8] o Developing meta-awareness* • *Mantra meditation*: creating stability of the mind without the need for intense focus [7, 8]
**Open-monitoring meditation**	• No explicit focus on objects [6, 8] • Observing anything that occurs in the current experience (sights, sounds, sensations, emotions, thoughts) [1••, 6, 8]	• Cultivating a non-reactive (reflexive) and non-judgmental awareness of cognitive and emotional perceptions [1••, 2••, 6, 8] • Cultivating meta-awareness or metacognition*
**Loving kindness and compassion meditation**	• Direct manipulation of thoughts and emotions through mental imagery techniques [8, 9]	• Cultivating feelings of kindness and compassion towards others [1••, 6, 10] • Shifting self-referential cognitive and affective patterns towards thoughts that involve the well-being of others [8]

*Meta-awareness or metacognition can be defined as being aware of the ongoing physical and self-referential processes of consciousness [6, 8, 9, 11]

The experience of pain is a complex construct and the exact neurobiological mechanisms through which mindfulness meditation can influence pain perception have until recently been poorly understood. Although meditation shares several characteristics with other self-regulation practices, such as psychotherapy or hypnosis—the two alternative treatment modalities for pain—these practices generally do not focus on training attention and awareness. Hypnotic techniques, as an example, generally influence mental contents on an unconscious level, such as through thoughts, images, or emotions, while meditation trains the ability to observe and control ongoing cognitive processes [3, 12]. After all, evidence regarding the use of meditation in relieving acute pain is still scarce. We present the findings of recent studies on the specific mechanisms of mindfulness meditation–induced pain relief. We describe specific brain regions that are involved in the cognitive processing and modulation of pain and the ways in which meditation interacts with these brain regions. We also summarize the current evidence provided by experimental and clinical studies that support the use of mindfulness meditation in patients suffering from acute pain and, based on these findings, we finally discuss how mindfulness meditation might be implemented in clinical practice.

## The Concepts of Consciousness and Pain Perception

From a neurobiological perspective, meditation can be thought of as a modification of consciousness [2••]. Consciousness remains a complex concept due to its multidimensionality and the uniqueness of each human experience, which is shaped by sensory, affective, and cognitive aspects. Simply defined, consciousness is a cognitive state in which one is aware of the individual experience and the surrounding environment [13, 14]. Consciousness is dictated by the presence of wakefulness (level of consciousness) and awareness (context of consciousness) [14]. Additionally, neurobiological research divides the states of consciousness into two classes: global and local states of consciousness [15]. Global states describe changes in arousal and behavioral responses, such as wakefulness, dreaming, and sedation. Local states involve sensory, affective, and cognitive content. They describe the “experience of self,” which includes experiences of mood, emotion, volition, body ownership, and explicit autobiographical memory [15].

The understanding of pain perception, just as the concept of consciousness, is based on a multidimensional model involving sensory, affective, and cognitive aspects [13]. The International Association for the Study of Pain (IASP) defines pain as “an unpleasant sensory and emotional experience associated with, or resembling that association with, actual or potential tissue damage” [16]. Although the processing of nociception is largely unconscious, the perception of pain requires a state of consciousness (wakefulness and awareness) that is influenced by psychological and cognitive factors [13, 14]. Traditional Buddhist texts have long suggested that pain perception is the result of both sensory and affective factors, and that trained meditators experience pain in a different way than untrained individuals. The original texts liken the physical and mental aspects of pain to being struck by two subsequential darts or arrows. While untrained individuals will be pierced by both arrows, experienced meditators will be able to influence their experience of pain by avoiding the second arrow, which represents the worry and distress caused by a painful event [7, 11, 12].

### A Synopsis of Pain Physiology

Pain physiology is influenced by a complex network of interactions involving the autonomic, peripheral, and central nervous systems [17, 18], as well as the endocrine [19] and immune systems [20, 21]. Initial nociceptive information is conducted along primary afferent fibers to the dorsal horn of the spinal cord [22, 23]. From here, the information ascends to the thalamus via the spinothalamic tract and to specific areas in the brainstem via the spinomesencephalic and spinoreticular tracts [24]. From the thalamus, nociceptive information is projected to the cortex, where cognitive processing integrates sensory-discriminative (i.e., location and intensity) and affective-emotional (i.e., pain unpleasantness) aspects [17, 25•]. Research using positron emission tomography (PET) and functional magnetic resonance imaging (fMRI) has unmasked a complex network of brain regions that are involved in the cognitive processing and top-down modulation of pain [17, 25•]. In Table 2, we list the relevant brain regions involved in pain processing and modulation. We also summarize the mechanisms through which mindfulness meditation influences pain perception.

**Table 2:** Overview of central nervous system areas of importance for pain physiology and modulating mechanisms of mindfulness meditation

**Central nervous system**	**Role in pain physiology**	**Mechanisms of pain modulation by mindfulness meditation**
**Spinal cord** - Dorsal horn	Transmits nociceptive information from primary afferent fibers to the brainstem (PAG, RVM) and thalamus [23]	
**Brainstem** - Periaqueductal gray (PAG) - Rostral ventral medulla (RVM)	Receives ascending nociceptive information from the dorsal horn and descending inhibitory projections from (sub)cortical regions (PFC, ACC, amygdala), which descend from the PAG via the RVM to the dorsal horn [17, 26, 27]	PAG deactivation through top-down inhibition in novice meditators [28]
**Thalamus**	Main relay center for ascending nociceptive information to subcortical and cortical brain regions [24]	Decreased activity through top-down inhibition in novice meditators [28-29] Reduced connectivity to S1 in novice meditators [30, 29]
**Somatosensory cortex (SSC)** - Primary sensory cortex (SI) - Secondary sensory cortex (SII)	Processes sensory-discriminative aspects of pain [27] Pain character and location (SI) [31, 32] Intensity of painful stimuli (SII) [33]	Reduced connectivity to the thalamus [30] Decreased activity in novice meditators [30, 34] Increased activity in experienced meditators [35, 36]
**Anterior cingulate cortex (ACC)**	Processes affective-emotional aspects of pain (i.e., pain unpleasantness) [27, 32, 37] Integration of emotional and cognitive modulation [38, 39], inhibitory projections to the brainstem (PAG) [27], attentional regulation [40••] and behavioral response to pain [41]	Increased activity in novice meditators [28, 30] Increased activity during pain anticipation in experienced meditators [35]
**Insula**	Processes affective-emotional aspects of pain (i.e., pain unpleasantness) [27, 30, 38] Convergence of extero- and interoceptive sensory information [42]	Increased activity in novice meditators [28, 30] and experienced meditators [35]
**Amygdala**	Integration of emotional and cognitive modulation [43], inhibitory projections to other subcortical regions (ACC, PAG) [17, 27, 43]	Decreased activity in experienced meditators [29, 36]
**Hippocampus**	Key structure for executive function [44], projections to the PAG [45]	Decreased activity in experienced meditators [29, 36]
**Prefrontal cortex (PFC)**	Cognitive processing of nociception (appraisal, contextual meaning) [26, 46] Attentional and emotional regulation [46, 47] Pain inhibition through descending connections to subcortical structures (thalamus, insula, ACC, PAG) [13, 27, 38, 47] Node of the default mode network (self-referential processing) [47-48]	Decreased activity in experienced meditators [35, 36] Increased activity in novice meditators [28]
**PCC/precuneus**	Node of the default mode network (self-referential processing) [40••, 49]	Deactivation in novice meditators [28] Increased connectivity to SSC [34] Reduced connectivity to the thalamus [29] Reduced synchronization between DMN nodes [34]

## Meditation and Pain Modulation

**Fig. 1:**
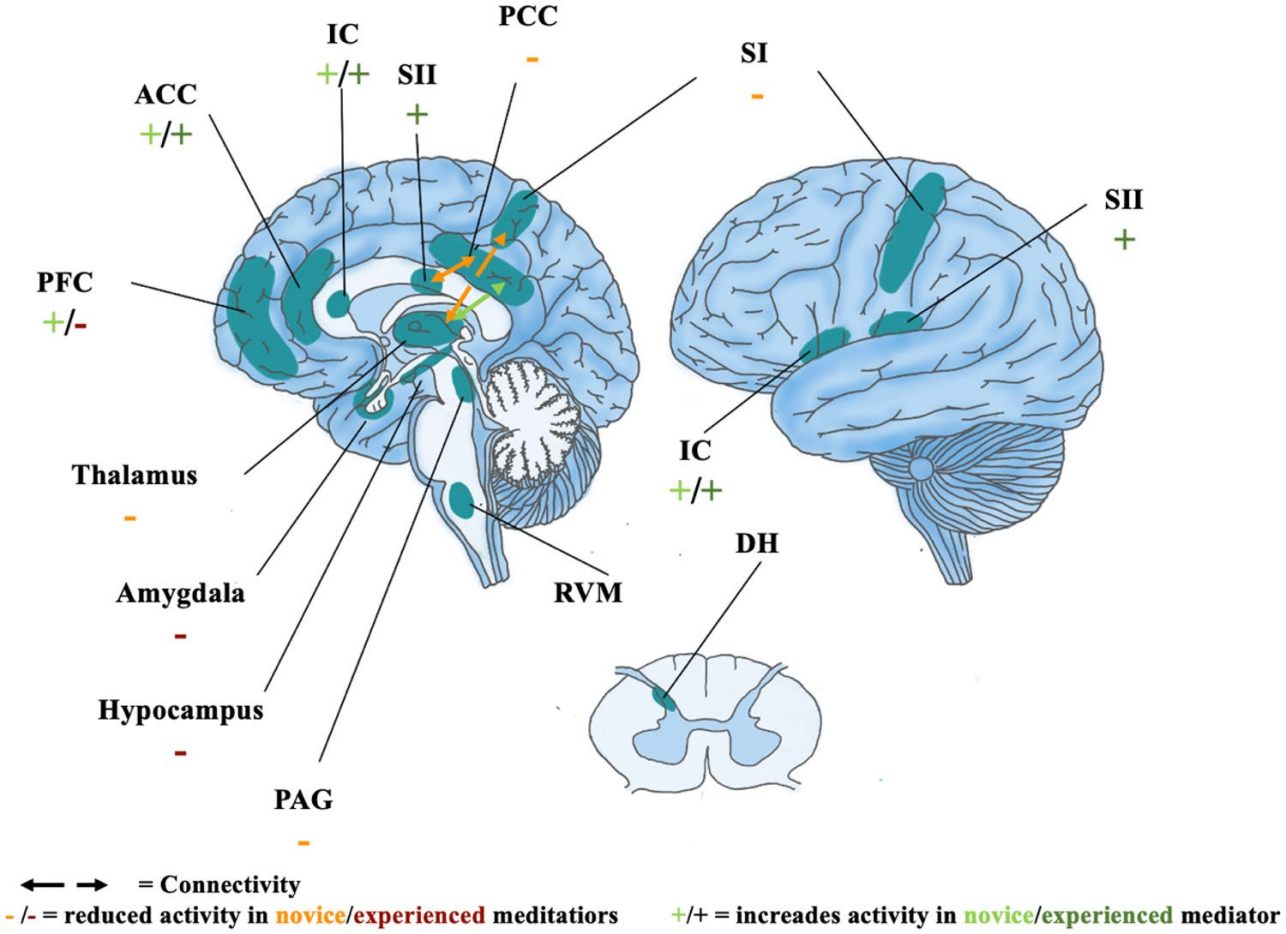
Brain regions involved in pain modulation by meditation (adapted from Tang, YY., Hölzel, B., and Posner, M. The neuroscience of mindfulness meditation. Nat Rev Neurosci 16, 213–225 (2015). https://doi.org/10.1038/nrn3916 [40••]). ACC anterior cingulate cortex, IC insula, PFC prefrontal cortex, SI primary sensory cortex, SII secondary sensory cortex, PCC posterior cingulate cortex, PAG periaqueductal gray, RVM rostral ventral medulla, DH dorsal horn

Neuroimaging studies have recently provided insight into the neurocognitive mechanisms by which mindfulness meditation can influence pain perception [25•, 26, 50••, 51]. Current research suggests that the mechanisms of mindfulness meditation–induced pain relief are different in novice and experienced meditators. Brief meditation training (less than 10 h of practice) seems to be associated with top-down modulation of ascending nociceptive information in the thalamus by projections coming from higher-order brain regions that are involved in the cognitive processing of pain (Fig. 1) [25•, 26]. For example, studies have shown that reductions in pain intensity and pain unpleasantness are associated with activation of the orbitofrontal cortex (OFC), the anterior cingulate cortex, and the anterior insula [28, 30]. Concurrently, there is a downregulation of signals in the thalamus and reduced activation of the area in the somatosensory cortex corresponding to the stimulation site [30]. This suggests the involvement of a cortico-thalamic gating mechanism, which reduces the thalamic transmission of nociceptive input to the somatosensory cortex [50••].

In addition, mindfulness meditation in inexperienced practitioners reduces activation of the precuneus, which is a central node of the default mode network (DMN) [49]. In neuroscience, the DMN refers to a network of brain regions that are involved in appraisal and self-referential processing. It is characterized by oscillating activity between these regions, with increased activity during wakeful resting states (e.g., daydreaming, mind wandering) and decreased activity during attention-demanding tasks [52] (Ricard 2014). Recent studies have shown that mindfulness meditation not only reduces synchronization between the nodes of the DMN [34], but also between the DMN and the thalamus [29]. At the same time, mindfulness meditation increases connectivity between the DMN and the somatosensory cortices [34]. These data suggest that mindfulness meditation not only reduces pain through cortico-thalamic inhibition, as mentioned above, but may also modulate pain perception through reappraisal of the incoming nociceptive information [25•, 26, 50••].

In contrast, experimental data has shown that long-term mindfulness meditation training (more than 1000 h of practice) primarily influences pain perception through a reduction in pain unpleasantness rather than pain intensity [35, 53, 54]. Reduced pain unpleasantness in experienced meditators is associated with significantly increased activity in somatosensory regions and simultaneous deactivation of appraisal-related brain regions [35, 36]. By focusing on the sensory-discriminative rather than the affective-emotional aspects of incoming nociceptive information, mindfulness meditation can induce a mental shift, which effectively uncouples the affective and sensory processing centers of pain [48, 54, 55]. Furthermore, researchers have recently developed machine-learning techniques, which identify distinct fMRI activity patterns, or neural signatures, that are associated with the processing of pain intensity and pain modulation [56, 57]. Although both short- and long-term meditation training is associated with subjective pain relief, the neural signatures of novice and experienced meditators of fMRI imaging differ significantly when they are subjected to a painful stimulus [58]. This provides further evidence that pain modulation depends on specific mechanisms which are developed and enhanced through practice and experience [26].

Importantly, the described mechanisms of mindfulness meditation–induced pain relief are independent of the endogenous opioid pathway [59-60] and occur through mechanisms that are distinct from placebo [28, 61, 62]. Although mindfulness meditation and sham (or placebo) meditation both reduce pain intensity and pain unpleasantness, the effect of sham meditation is less pronounced. Healthy subjects performing sham meditation also show greater activation in key regulatory brain regions such as the thalamus and the DMN, which are downregulated in subjects performing mindfulness meditation [28]. Moreover, an infusion of naloxone does not reverse mindfulness meditation–induced reductions in pain intensity and pain unpleasantness [59]. Although the exact mechanism is unknown, naloxone-mediated blockade of opioid receptors can even enhance the effect of mindfulness meditation on pain intensity scores [63, 62]. Additionally, recent findings have implicated the autonomic nervous system as a mediator for mindfulness meditation–induced pain relief. A higher heart rate variability during mindfulness meditation, as a marker for increased parasympathetic activity, is strongly associated with lower pain unpleasantness when compared to sham meditation [61]. Although the precise mechanism of autonomic pain modulation remains unclear [18], these findings are in line with the idea that mindfulness meditation engages neural mechanisms that are distinct from placebo to reduce pain [64–67].

## Meditation as a Remedy for Acute Pain

One of the main challenges in meditation research has been the lack of a standardized classification system for mindfulness-based interventions (MBIs) [35, 54]. Clinical research on MBIs has mostly been based on methods first developed and described by Jon Kabat-Zinn and colleagues more than 40 years ago [68, 69]. Since then, much research has been devoted to the effect of MBIs in patients with chronic pain [70]. Despite substantial heterogeneity among the available data, there is now consensus that MBIs can have a positive effect on pain intensity and the quality of life in patients with chronic pain [70, 71]. In acute pain research, the effect of MBIs has mainly been examined in healthy volunteers in experimental settings, although there has recently been an increase in clinical studies [72]. Experimental data show that even brief periods of provider-led mindfulness meditation training (3–4 days) in inexperienced subjects can significantly reduce pain intensity and pain unpleasantness [28, 30, 73]. A 20-min training session, followed by 2 weeks of self-practice at home, has also been found to increase pain thresholds and provide a more rapid attenuation of pain intensity in healthy volunteers [74]. Since meditation training is usually time intensive, the potential benefits of such brief MBIs provide new perspectives for the integration of meditation in everyday clinical practice [75].

Current research suggests that brief MBIs can be successfully implemented in hospitalized patients suffering from anxiety and acute pain and in the perioperative setting [76]. In hospitalized patients with either “intolerable pain” or “inadequate pain control,” approximately one-third of the patients treated with a single 15-min MBI reported at least a 30% reduction in pain intensity, which is comparable to a dose of 5 mg oxycodone [48]. Similarly, in patients admitted to a surgical center with acute pain due to different medical problems (e.g., infection, bowel obstruction, trauma), a 10-min MBI was able to significantly reduce pain intensity and pain-related stress [77]. In patients planned for total knee or hip joint arthroplasty, a single 15-min MBI reduced preoperative pain intensity from degenerative joint disease by 27%. In addition, patients showed reductions in pain unpleasantness, anxiety, and the desire for pain medication. Moreover, patients in the MBI group showed significantly better physical function at the 6 weeks postoperative evaluation [78]. As some authors have demonstrated, the preoperative evaluation of surgical patients provides an excellent opportunity to offer professional instruction and distribute educational materials for later self-practice [76, 79].

Furthermore, MBIs have been associated with beneficial outcomes for patients undergoing painful medical procedures. In dental surgery, 30–40 min of preoperative heartfulness meditation was associated with reduced intraoperative anxiety (although it had no effect on intraoperative pain intensity) [80]. In women undergoing stereotactic breast biopsy, studies employing mindfulness meditation and loving kindness meditation have shown mixed results on intraoperative pain intensity [81-82]. On the other hand, these studies also demonstrated that meditation provides relief from intraoperative anxiety and discomfort. Additionally, although a weekend mindfulness meditation course did not improve labor pain or reductions in epidural use in pregnant women going through childbirth, it did lower the need for opioid analgesia during labor [83]. An opioid-sparing effect has also been suggested by a recent meta-analysis evaluating MBIs in patients with clinical pain managed by opioids, although the study was not specific to acute pain [84]. Therefore, future research investigating MBIs as a remedy for acute pain might demonstrate further opportunities to treat acute pain in different clinical contexts and possibly contribute to the prevention of postoperative chronic pain and opioid misuse [85].

## Conclusion

Meditation-supported pain relief is based on changes in neurocognitive processes that can be identified through neuroimaging studies. The mechanisms through which meditation influences pain modulation evolve with practice and experience. Although the benefits of meditation for patients with pain have been well-established, current evidence for the treatment of acute pain is just about to emerge and supports meditation as a promising perspective for the treatment of patients suffering from acute pain in various clinical settings.

## Data Availability

Not applicable.
